# Ferrum Dialysatum

**Published:** 1877-12

**Authors:** L. P. Yandell

**Affiliations:** Professor of Therapeutics and Clinical Medicine, University of Louisville


### FERRUM DIALYSATUM.

BY L. P. YANDELL, JR., M. D., Professor of Therapeutics and Clinical Medicine, University of Louisville.

Dialysed iron has been used to a limited extent for some years in Europe, but it is only within the past few months that it has attracted much attention in America. It is now manufactured on a large scale by Wyeth & Brother, of Philadelphia, and according to the highest authorities in pharmacy this American preparation is greatly superior to the foreign article.

For more than a month I have employed dialysed iron in my practice to the exclusion of all other forms of the metal, and with most gratifying results. The manufacturers claim for dialysed iron the following advantages: It is tasteless, does not blacken the teeth, does not produce headache, gastric derangement, or constipation, and acts as efficaciously in ten-drop doses given four or five times a day as in double or quadruple these quantities. Besides, it is asserted to be equally as reliable an antidote to arsenic as the hydrate sesquioxide. In color it is a bright, reddish brown. Taken undiluted it has the faintest possible styptic taste, but this is not observable when water is added. It is possessed of a slight, peculiar flavor, resembling that of fresh blood, but not in the least unpleasant. In cold weather it is said to become thick—gelatiniform, and that a few drops of impure water to a considerable quantity of the fluid may produce this result at any time. This coagulation I have noticed even with the thermometer ranging between 80° and 90°; but a small quantity of distilled water added immediately restores its liquidity.

In anæmia, from various causes, in the neuroses, dermatoses, menstrual derangements, phthisis, syphilis, sexual debility, and indeed in all of the morbid conditions for which I have prescribed *ferrum dialysatum* it has given perfect satisfaction, except in two cases, where it produced some constipation; but in both these instances it was administered in what the manufacturers state are unnecessarily large doses. In one, a lady, half teaspoonful was given thrice daily, and in the other, a girl of six years, fifteen drops were given thrice daily. In many others, half-teaspoonful doses given three to six times in the twenty-four hours led to no unpleasant results. In restoring the appetite, and in building up strength this form of iron has seemed to surpass all others. Already Messrs. Wyeth's new preparation has become popular with those who have used it, and when its excellencies have become generally known, it is safe to predict that it will supersede nearly all of the other ferruginous preparations.

Anstie insisted on the necessity of iron in the treatment of the neuroses, and what he claimed for iron in this class of maladies is equally true of other diseases. Iron deserves to rank as the king of metals in medicine, as it does in the arts and manufactures.

Dialysed iron may be given in any vehicle desired—syrups, elixirs, tinctures, or glycerites, but simple water is most likely to be preferred by the sick, as in this way it is without taste, and sick people usually dislike flavors.

Many formulæ are suggested in the pharmaceutical journals for the manufacture of dialysed iron, but with its mode of production the physician is not concerned. Suffice it to say that this addition to the materia medica is made from the liquor of the perchloride of iron, and the dialysis is performed by percolation through parchment, bladder, or fine parchment paper, after the components of the medicine have been properly commingled.—*Louisville Medical News.*

